# The impact of bilateral versus unilateral anterior temporal lobe damage on face recognition, person knowledge and semantic memory

**DOI:** 10.1093/cercor/bhae336

**Published:** 2024-08-10

**Authors:** Matthew A Rouse, Siddharth Ramanan, Ajay D Halai, Angélique Volfart, Peter Garrard, Karalyn Patterson, James B Rowe, Matthew A Lambon Ralph

**Affiliations:** MRC Cognition and Brain Sciences Unit, University of Cambridge, 15 Chaucer Road, Cambridge CB2 7EF, UK; MRC Cognition and Brain Sciences Unit, University of Cambridge, 15 Chaucer Road, Cambridge CB2 7EF, UK; MRC Cognition and Brain Sciences Unit, University of Cambridge, 15 Chaucer Road, Cambridge CB2 7EF, UK; Université de Lorraine, CNRS, 2 avenue de la Forêt de Haye, Nancy F-54000, France; Psychological Sciences Research Institute, University of Louvain, Place du Cardinal Mercier, 10, Louvain-la-Neuve B-1348, Belgium; School of Psychology and Counselling, Faculty of Health, Queensland University of Technology, Victoria Park Road, Brisbane 4059, Australia; Molecular and Clinical Sciences Research Institute, St George’s, University of London, Cranmer Terrace, London SW17 0RE, UK; MRC Cognition and Brain Sciences Unit, University of Cambridge, 15 Chaucer Road, Cambridge CB2 7EF, UK; Department of Clinical Neurosciences, University of Cambridge, Hills Road, Cambridge CB2 0SZ, United Kingdom; MRC Cognition and Brain Sciences Unit, University of Cambridge, 15 Chaucer Road, Cambridge CB2 7EF, UK; Department of Clinical Neurosciences, University of Cambridge, Hills Road, Cambridge CB2 0SZ, United Kingdom; Cambridge University Hospitals NHS Foundation Trust, Hills Road, Cambridge CB2 0SZ, United Kingdom; MRC Cognition and Brain Sciences Unit, University of Cambridge, 15 Chaucer Road, Cambridge CB2 7EF, UK

**Keywords:** face recognition, person knowledge, prosopagnosia, semantic dementia, temporal lobe epilepsy

## Abstract

The functional importance of the anterior temporal lobes (ATLs) has come to prominence in two active, albeit unconnected literatures—(i) face recognition and (ii) semantic memory. To generate a unified account of the ATLs, we tested the predictions from each literature and examined the effects of bilateral versus unilateral ATL damage on face recognition, person knowledge, and semantic memory. Sixteen people with bilateral ATL atrophy from semantic dementia (SD), 17 people with unilateral ATL resection for temporal lobe epilepsy (TLE; left = 10, right = 7), and 14 controls completed tasks assessing perceptual face matching, person knowledge and general semantic memory. People with SD were impaired across all semantic tasks, including person knowledge. Despite commensurate total ATL damage, unilateral resection generated mild impairments, with minimal differences between left- and right-ATL resection. Face matching performance was largely preserved but slightly reduced in SD and right TLE. All groups displayed the familiarity effect in face matching; however, it was reduced in SD and right TLE and was aligned with the level of item-specific semantic knowledge in all participants. We propose a neurocognitive framework whereby the ATLs underpin a resilient bilateral representation system that supports semantic memory, person knowledge and face recognition.

## Introduction

The role of the anterior temporal lobes (ATLs) has become of key interest to cognitive neuroscientists in recent years, resulting in two very active but largely distinct research pursuits. There is evidence from neuropsychology and functional neuroimaging that the ATLs are important for face recognition/person knowledge (Haxby et al. 2000; Gobbini and Haxby 2007; Collins and Olson 2014a; Duchaine and Yovel 2015). A separate literature implicates the ATLs in multimodal semantic memory, including knowledge of familiar faces/people alongside all other concepts (Patterson et al. 2007; Lambon Ralph et al. 2017). These two research areas and associated theories have remained largely separate from each other, despite making potentially complementary predictions. The aim of the current study was to bridge the two literatures and generate a unified neurocognitive framework for the role of the ATLs in face recognition, person knowledge and semantic processing. Accordingly, a bespoke neuropsychological battery was used to assess the effect of bilateral vs. unilateral ATL damage on (i) general semantic memory vs. person knowledge and (ii) perceptual face matching of famous and unfamiliar faces. To identify the effects of bilateral vs. unilateral (left vs. right) ATL damage, we compared two patient groups associated with ATL damage; semantic dementia (SD, encompassing semantic variant primary progressive aphasia and “right” SD), associated with bilateral ATL atrophy from neurodegeneration (Neary et al. 1998; Gorno-Tempini et al. 2011), and people who had undergone left or right unilateral ATL resection for temporal lobe epilepsy (TLE).

There is evidence from positron emission tomography (Sergent et al. 1992; Gorno-Tempini et al. 1998; Kuskowski and Pardo 1999; Gorno-Tempini and Price 2001), functional magnetic resonance imaging (fMRI) (Leveroni et al. 2000; Kriegeskorte et al. 2007; Rajimehr et al. 2009; Nasr and Tootell 2012), and intracranial electrode recordings (Allison et al. 1999) that the ATLs respond to familiar faces, as well as neuropsychological demonstrations of impaired face recognition following ATL damage from neurodegenerative disorders or unilateral resection (Evans et al. 1995; Glosser et al. 2003; Drane et al. 2013; Hutchings et al. 2017; Borghesani et al. 2019; Ding et al. 2020). Based on these findings, neurocognitive models of face recognition have broadened to include the ATL as part of an extended network critical for linking faces with stored semantic knowledge (Haxby et al. 2000; Gobbini and Haxby 2007; Collins and Olson 2014a; Duchaine and Yovel 2015). Indeed, the existence of face-selective patches in the ATL has been proposed (Rajimehr et al. 2009; Collins and Olson 2014a), thought to be homologous to the anterior face patches identified in macaques (Pinsk et al. 2005; Tsao et al. 2006; Hesse and Tsao 2020).

In contrast to a face-specific function, there is convergent evidence from neuropsychology, fMRI, transcranial magnetic stimulation (TMS) and grid electrode studies that the ATLs are critical for supporting semantic memory more broadly (Mummery et al. 2000; Lambon Ralph et al. 2009; Binney et al. 2010; Shimotake et al. 2015; Rogers et al. 2021). Perhaps most strikingly, people with SD, associated with bilateral ATL atrophy, display a global degradation of conceptual knowledge (Snowden et al. 1989; Hodges et al. 1992; Mummery et al. 2000; Gorno-Tempini et al. 2011). This semantic degradation occurs for all types of concepts, including but not limited to knowledge of familiar faces/people (Mummery et al. 2000; Snowden et al. 2004; Patterson et al. 2007; Snowden et al. 2012; Lambon Ralph et al. 2017). Based on these findings, the ATLs have been considered to underpin a semantic hub critical for the creation of generalizable concepts from the numerous multimodal experiences we have of each concept over our lifetimes (Rogers et al. 2004a; Patterson et al. 2007; Lambon Ralph et al. 2017). To achieve this, the hub interacts with modality-specific cortical “spokes” and integrates multimodal information across experiences to distil coherent concepts. Thus the hub is transmodal and trans-category as it supports the activation of semantic representations across all modalities and semantic categories (Patterson et al. 2007; Lambon Ralph 2014; Lambon Ralph et al. 2017). The hub is also crucial for the integration of information across time and contexts, allowing for the “transtemporal” extraction of semantic structure (Jackson et al. 2021).

FMRI studies consistently detect strong bilateral ventrolateral ATL activation in relation to all types of conceptual knowledge (Binney et al. 2010), as long as techniques are utilized to maximize ATL signal (Halai et al. 2014). A recent study found that this same region activated in response to both the faces and spoken/written names of famous people and to specific-level concepts other than people, such as famous landmarks. An anterior extension to this core ventrolateral ATL region demonstrated weaker yet more selective activation for people over the other categories (overlapping with the peaks described in the face recognition literature) (Rice et al. 2018c).

ATL damage does not generate the perceptual face processing deficits associated with damage to posterior temporal cortex (e.g. the fusiform face area) (Barton et al. 2002; Barton 2008). People with SD perform at normal levels on tasks of perceptual matching of unfamiliar faces, which require distinguishing between faces but do not require activation of specific conceptual knowledge (Hutchings et al. 2017; Borghesani et al. 2019). Healthy participants match famous/familiar faces faster and more accurately than unfamiliar faces (Bruce 1986; Young et al. 1986; Bruce et al. 2001; Clutterbuck and Johnston 2002; Megreya and Burton 2006; Visconti et al. 2017). This has led some researchers to argue for qualitative differences in how familiar and unfamiliar faces are processed (Megreya and Burton 2006). One potentially important difference is that familiar faces are laden with specific semantic knowledge, which may support and facilitate face processing, whereas perception of unfamiliar faces can only rely on visual features (Collins and Olson 2014b; Rossion 2018). Therefore, although not *critical*, the ATLs may enhance performance in tasks that require perceptual matching of faces through feedback activation, thus reducing perceptual demands and contributing to the known familiarity effect (Collins and Olson 2014b; Rossion 2018). There is behavioral evidence for an interaction between perceptual and conceptual information in healthy participants, where associating previously unfamiliar stimuli with conceptual labels improves later recognition (Lupyan and Spivey 2008; Schwartz and Yovel 2016), and from people with SD who are impaired on tasks which require successfully classifying visually different exemplars of objects as the “same thing,” or words/objects as real vs. nonreal (Rogers et al. 2003; Rogers et al. 2004b; Ikeda et al. 2006).

The differential function of the left and right ATLs is a key area of debate (Gainotti 2015). Face recognition theories have generally made no strong claims regarding left/right ATL differences (Haxby et al. 2000; Gobbini and Haxby 2007); however, the early stages of face perception in ventral occipital-temporal regions are thought to be supported bilaterally with a right-sided dominance (Kanwisher et al. 1997; Ishai et al. 2005; Rossion et al. 2012; Behrmann and Plaut 2015). Neuropsychological studies have implicated the importance of the right ATL in face recognition, based on several case reports of prosopagnosia after right ATL damage from either SD or unilateral resection (Evans et al. 1995; Seidenberg et al. 2002; Glosser et al. 2003; Joubert et al. 2003; Chan et al. 2009). The underlying explanation for this right ATL bias is debated, with suggestions that the right ATL is specialized for representing multimodal person-specific semantic knowledge, rather than faces specifically (Younes et al. 2022) or alternatively that the right ATL is specialized for retrieving semantic information from visual inputs (i.e. faces) whereas the left ATL is important for retrieving verbal semantics (e.g. written and spoken names) (Snowden et al. 2004, 2012). The evidence from functional neuroimaging is less clear cut, with evidence for bilateral ATL activation in response to faces or people’s names (Rice et al. 2015b; Rice et al. 2018c).

A hub-and-spoke model of semantic memory has been proposed in which the bilateral ATLs work in concert to support transmodal semantic representations, and that bilateral neural implementation can make functional systems more resilient to unilateral damage (Schapiro et al. 2013; Jung and Lambon Ralph 2016; Lambon Ralph et al. 2017). This framework does not deny emergent functional differences between the left and right ATLs but, in line with various computational modeling demonstrations, suggests that these differences could be a consequence of differential connectivity of the left/right ATL with modality-specific cortical regions (Lambon Ralph et al. 2001; Behrmann and Plaut 2015; Rice et al. 2015a; Woollams and Patterson 2018). The right posterior temporal cortex is more dominant for face processing (Behrmann and Plaut 2015; Hoffman and Lambon Ralph 2018) and so consequently the face recognition problems associated with right ATL damage may be because the right ATL receives increased visual input from right posterior temporal areas (Hoffman and Lambon Ralph 2018).

To determine the impact of bilateral vs. unilateral damage and the relative contributions of the left/right ATL to semantics and face recognition, we directly compared people with SD to people with unilateral ATL resection, using the same neuropsychological and structural imaging measures. Although ATL abnormalities can be somewhat asymmetric in SD patients, especially initially, there is always hypometabolism and indeed some atrophy in the contralateral ATL (Nestor et al. 2006; Ding et al. 2020); and with progression of the disease, damage on the initially less affected side catches up (Brambati et al. 2009). Furthermore, the volume loss in SD is not restricted to the ATLs; atrophy also occurs in posterior temporal and prefrontal cortical regions as the disease spreads (Rosen et al. 2002; Gorno-Tempini et al. 2004). In contrast to SD, unilateral ATL resection provides a neuroanatomical model of purely unilateral ATL resection and the individual contributions of the left and right ATL (Lambon Ralph et al. 2012; Rice et al. 2018a). FMRI in ATL-resected TLE participants has demonstrated upregulation in the intact contralateral ATL during semantic tasks, which suggests that the undamaged ATL is able to maintain residual semantic performance (Rice et al. 2018b).

## Materials and methods

### Participants

Sixteen people with SD were recruited from specialist neurology clinics at Addenbrooke’s Hospital, Cambridge (*n* = 11), John Radcliffe Hospital, Oxford (*n* = 4) and St George’s Hospital, London *(n* = 1). All SD patients met diagnostic criteria for SD (Neary et al. 1998). Seventeen people who had undergone unilateral en bloc anterior temporal lobectomy for TLE (left TLE = 10, right TLE = 7) were recruited from neuropsychology departments at Salford Royal NHS Foundation Trust, Manchester (*n* = 8) and Walton Centre NHS Foundation Trust, Liverpool (*n* = 9). All the ATL-resected cases had had late-onset, long-standing TLE stemming from unilateral hippocampal sclerosis, were left language dominant based on Wada testing and were at least 12 months postsurgery. Normative data were obtained from fourteen healthy volunteers with no history of neurological or psychiatric disorders, recruited from the MRC Cognition and Brain Sciences Unit, University of Cambridge. All participants provided written informed consent under approval by the National Research Ethics Service Committee.

### General semantic memory

General semantic memory was assessed using a battery of receptive and expressive tasks using verbal and pictorial stimuli. Tasks included the modified picture-version of Camel and Cactus semantic association test (mCCT) (Bozeat et al. 2000; Moore et al. 2022), a synonym judgment task (Jefferies et al. 2009; Halai et al. 2022), the Cambridge Naming (Bozeat et al. 2000; Halai et al. 2022) and Boston Naming (Kaplan et al. 2001; Halai et al. 2022) tests and a word-to-picture matching task (Rouse et al. 2024). For all patients (*n* = 33), a principal component analysis (PCA) was conducted on the mCCT, synonym judgment task and word-to-picture matching task scores. The PCA generated one component with an eigenvalue greater than one (2.69) which explained 89.5% of the total variance (Kaiser-Meyer-Olkin statistic = 0.68). All three tasks loaded heavily onto this component (mCCT = 0.92, synonym judgment task = 0.94, word-to-picture matching task = 0.98), and so factor scores were used as a composite score of total semantic impairment. The lower bound of normality for the composite score was derived by calculating the factor score of a hypothetical individual scoring 1.96 standard deviations below the control mean on all three tasks. Global cognitive function was assessed using the Addenbrooke’s Cognitive Examination-Revised (Mioshi et al. 2006) and executive functioning assessed using the Brixton Spatial Anticipation Test (Burgess and Shallice 1997) and Raven’s Colored Progressive Matrices B (Raven 1962).

### Person knowledge

Two 44-item tasks were designed to assess person knowledge: face-to-name matching and face-to-profession matching. In each task, participants were shown a photograph of a famous person and instructed to point to the correct name/profession from four possible response options. In the face-name matching task, distractor items were the names of other famous people with the same gender and similar occupation. In the face-profession matching task, distractor items were alternative professions. Participants also completed a difficulty matched 42-item landmark-to-name matching task, to determine whether any person knowledge deficits were selective, or occurred for another type of specific-level concept. Participants were shown a photograph of a famous landmark (e.g. Taj Mahal) and were asked to point to the correct name from four possible response options, where the distractor items were the names of alternative famous landmarks. Performance accuracy on the person knowledge tasks was negatively correlated with age in controls (face-name matching; *r* = −0.63, *P* < 0.05, face-profession matching; *r* = −0.56, *P* < 0.05) and so groups were compared using Quade’s nonparametric ANCOVA and post hoc Tukey’s range tests. Individual patient deficits were determined using one-tailed modified t-tests. This method tests whether an individual’s score on a task is significantly below a control sample, and is recommended when comparing against small control samples (Crawford and Howell 2010). For the two person knowledge tasks, single case deficits were tested using Bayesian methods to control for the effect of age (Crawford et al. 2011).

### Perceptual face matching

A face matching task (Volfart et al. 2022) was administered on a laptop using E-prime software (version 1.2, Psychology Software Tools). Participants were presented with a triad of faces; one at the top of the screen and two below and were instructed to select which of the two faces below was the same person as the face at the top. Participants performed practice trials to ensure they understood the task, and accuracy and response times (RT) were recorded. There was no time limit, but participants were instructed to respond as quickly as they could. There were 44 items in total, where half of the faces were famous (using people from the person knowledge tasks as targets or foils) and half were unfamiliar. Only trials receiving correct responses were included in the RT analysis. Outlier RTs for each participant (1.96 standard deviations away from the participant’s mean RT) were replaced by their mean RT across all conditions (Lavallee et al. 2016).

Numerous investigations of face processing in healthy participants have included and utilized a presentation manipulation known as the “inversion effect.” Accordingly, we included the same manipulation in this neuropsychological exploration. Faces are much more difficult to recognize when they are upside down compared to upright, and this “inversion effect” is disproportionately larger for faces than for other visual object categories (Yin 1969; Valentine 1988). This phenomenon has led to proposals that faces are processed holistically, and that inversion disrupts holistic/configural processing meaning that identification/recognition must rely more on feature-by-feature comparisons (Freire et al. 2000). We explored whether inversion eradicated the familiarity effect, or whether conceptual knowledge boosted recognition even when famous faces were inverted. Therefore, each face was presented both upright and inverted on separate occasions.

To assess the relationship between semantic knowledge and perception, each participant’s person knowledge results were used to categorize their face perception trials into “fully known” (correct in both person knowledge tasks), “partially known” (correct in only one person knowledge task) or “unknown” (incorrect in both person knowledge tasks). Friedman tests and post hoc Wilcoxon tests (corrected for multiple comparisons using the Holm method) were conducted to compare RTs across different levels of semantic knowledge and to unfamiliar face matching RTs.

### Magnetic resonance imaging

Thirty participants (16 SD, 14 controls) had a 3 T structural MRI scan on a Siemens PRISMA at the MRC Cognition and Brain Sciences Unit or the Wolfson Brain Imaging Centre (both in Cambridge). The TLE group’s structural MRI scans on a 3 T Phillips Achieva scanner were available from a previous study (Rice et al. 2018a; Rice et al. 2018b). One TLE participant had undergone further ipsilateral temporal neurosurgery since his scan and so was excluded from the imaging analysis. MRI scans from 20 controls scanned for the original TLE study were included in the imaging analysis, so that TLE groups could be compared to a group matched for both age and scanning site (Rice et al. 2018a; Rice et al. 2018b).

Voxel-based morphometry (VBM) was conducted to determine gray matter volumetric differences between patient groups and controls. A separate general linear model was created for each patient group vs. controls, with age, intracranial volume, and scanning site included as covariates. An explicit mask was used which excluded any voxels for which > 20% of participants had an intensity value of < 0.1; this is a method recommended for analysis of atrophic brains (Ridgway et al. 2009). Significant clusters were extracted using a voxelwise statistical threshold of *P* < 0.05 (FWE-corrected) with a cluster threshold of 100 voxels. To visualize both (i) the total amount and (ii) the distribution of ATL volume loss, gray matter intensities in the left and right ATL were extracted for each participant, using binarized masks derived from a previous ALE meta-analysis (Rice et al. 2015b). For each patient, values were z-scored relative to the control sample to calculate two indices; (i) ATL magnitude (left ATL volume + right ATL volume) and (ii) ATL asymmetry (left ATL volume—right ATL volume) (Borghesani et al. 2020; Ding et al. 2020; Ramanan et al. 2023; Rouse et al. 2024). Structural MRI is insensitive to some markers of neurodegeneration such as hypometabolism (Nestor et al. 2006) and synaptic loss (Malpetti et al. 2023), and thus VBM may underestimate the degree of ATL damage in SD. However, this caveat applies to unilateral resection too, where there may be additional damage secondary to the site of resection, such as white matter connectivity changes consistent with Wallerian degeneration (Wieshmann et al. 1999; Busby et al. 2019).

## Results

### Demographic and clinical information

Table 1 displays demographic and clinical information for participants. Groups were matched for sex and years of education. In keeping with the inherent aetiological differences, both TLE groups were younger on average than the neuropsychology controls and SD (*P* < 0.0001). The right TLE group had longer postresection durations than left TLE (*P* = 0.01). However, both groups averaged over ten years since resection meaning that any postsurgical plasticity-related differences between the groups are unlikely.

**Table 1:** Demographic and clinical information.

	Control	Left TLE	Right TLE	SD	Group effect	Effect size	Post-hoc tests
N	14	10	7	16	-	-	-
Sex (F:M)	7:7	5:5	4:3	9:7	4	V = 0.07^*^	-
Age (years)	64.1 (7.5)	45.2 (10.6)	53.1 (9.7)	65.2 (7.8)	**F(3,43) = 13.9, *P* < 0.0001**	η^2^ = 0.49†	L, R < C, SD
Education (years)	15.6 (3.3)	13.4 (2.8)	13.7 (2.1)	13.9 (2.9)	F(3,43) = 1.4, *P* = 0.25	η^2^ = 0.09†	-
Years since symptom onset	-	-	-	5.5 (3.3)	-	-	-
Years since surgery		10.9 (3.9)	15.9 (2.4)	-	**t(15) = 3.0, *P* = 0.01**	d = 1.52‡	-
Resection volume (mm^3^)	-	37.6 (10.0)	73.7 (20.1)	*-*	**t(15) = 4.8, *P* = 0.0003**	d = 2.27‡	-
Number of antiseizure drugs	-	2.2 (1.4)	1.6 (1.3)	*-*	t(15) = 0.98, *P* = 0.34	d = 0.49‡	-
ATL magnitude	-	−5.0 (2.4)	−9.7 (2.9)	−9.3 (2.9)	**F(2,29) = 8.3, *P* = 0.001**	η^2^ = 0.36†	L < R, SD
ATL asymmetry (absolute value)	-	27.3 (6.9)	34.1 (4.1)	6.3 (3.1)	**F(2,29) = 110.6, *P* < 0.0001**	η^2^ = 0.88†	SD < L, R

ATL = anterior temporal lobe, C = control, L = left TLE, R = right TLE, SD = semantic dementia, TLE = temporal lobe epilepsy.

†partial η^2^: small = 0.01, medium = 0.06, large = 0.14.

‡Cohen’s d: small = 0.2, medium = 0.5, large = 0.8.

a Cramer’s V: small = 0.1, medium = 0.3, large = 0.5.

### Magnetic resonance imaging

**Fig. 1:**
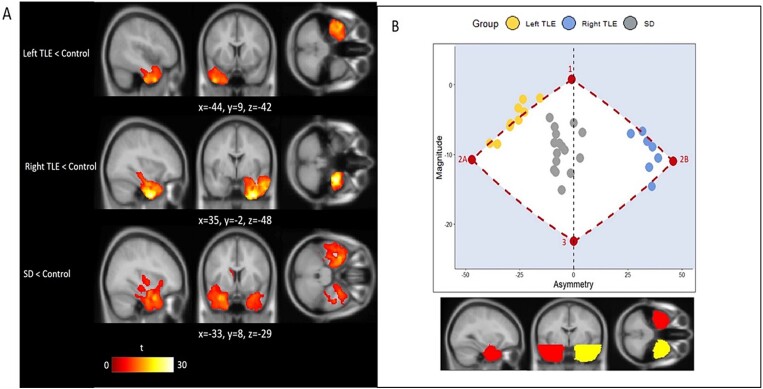
**Neuroimaging results.** (**A**) Voxel-based morphometry results showing regions of reduced gray matter volume in (i) left TLE, (ii) right TLE, and (iii) SD. Images are thresholded using a threshold of *P*(FWE) < 0.05 with a cluster threshold of 100 voxels. Significant clusters are overlaid on the MNI avg152 T1 template. Co-ordinates are reported in Montreal neurological institute space. (**B**) Scatter plot displaying ATL magnitude and asymmetry indices for each patient. Lower magnitude values indicate greater volume loss, and negative asymmetry values indicate left > right ATL volume loss. The points forming a diamond represent markers showing from (i) 1-2A/B, the extremity boundary after purely unilateral left/right resection; and (ii) 2A/B-3 being the most extreme one could be with additional levels of bilateral damage; until (iii) 3—Complete bilateral resection. The anatomical location of the left and right ATL masks used for deriving the magnitude and asymmetry indices is displayed below the scatter plot.

VBM was conducted to determine the location and extent of gray matter volume reduction in each patient group relative to age-matched controls. As expected, the SD group had significantly reduced gray matter in the bilateral ATLs. In contrast, each TLE group had one cluster of volume loss, in either the left or right ATL depending on the site of the neurosurgery (Fig. 1A and Supplementary Table 1). Individual ATL indices were calculated to directly compare SD and TLE groups on total ATL volume loss and asymmetry of ATL volume loss. Magnitude and asymmetry indices are displayed in Fig. 1B. There were overlapping levels of ATL magnitude between SD and TLE; the SD and right TLE patients were matched on ATL magnitude (*P* = 0.96), whereas the left TLE cases had higher magnitude indices (i.e. greater ATL volume) than both SD (*P* < 0.01) and right TLE (*P* < 0.01) (Table 1). The difference in ATL magnitude between left and right TLE is in keeping with current surgical standards, where resections of the left ATL are more conservative to avoid disruption to language networks (Wiebe et al. 2001). Despite similar levels of ATL magnitude, there was a large difference in ATL asymmetry between SD and TLE (*F*(2,29) = 110.6, *P* < 0.0001, η^2^ = 0.88). Although most of the SD group were asymmetric to a degree (with most having left > right damage), this was far lower than in TLE, highlighting the bilateral atrophy in SD. Significant differences in ATL asymmetry were found between SD and left TLE (*P* < 0.0001) and between SD and right TLE (*P* < 0.0001). Although each TLE group had high levels of asymmetry, the right TLE group was more asymmetric on average than left TLE (*P* < 0.05) reflecting the larger resection volumes.

### Semantic memory

Scores on each semantic task are reported in Table 2. Despite comparable volumes of overall ATL damage, the SD patients (bilateral damage) had considerably worse scores across the full range of semantic tasks than either TLE group or age-matched controls Generally, the left and right TLE groups were mildly impaired, with no left vs. right differences. The comparisons between TLE and controls that reached statistical significance after correcting for multiple comparisons were between left TLE and controls on the Boston Naming task (*P* < 0.05), Camel and Cactus (*P* < 0.05) and synonym judgment task (*P* < 0.01). In addition, the majority of the TLE sample (both left and right) (70.6%) had a semantic composite score below the control-derived lower bound of normality, consistent with the presence of a mild, global semantic impairment. P-values for each of the post hoc tests are reported in Supplementary Table 2.

**Table 2:** Neuropsychology scores.

	Control	Left TLE	Right TLE	SD	Group effect	Effect size	Post-hoc tests
N	14	10	7	16	-	-	-
ACE-R Total (100)	97.6 (1.3)	80.3 (10.4)	87.7 (6.1)	55.3 (15.2)	**F(3,15) = 50.7, P < 0.0001**	η^2^ = 0.75^*^	L, R, SD < C SD < L, R
MMSE (30)	29.8 (0.4)	27.3 (1.6)	28.9 (1.1)	22.9 (4.5)	**H(3) = 32.1, P < 0.0001**	η^2^ = 0.68^*^	L, SD < C SD < R
ACE-R Attention (18)	17.9 (0.3)	17.5 (0.8)	17.9 (0.4)	14.9 (3.0)	**H(3) = 23.6, P < 0.0001**	η^2^ = 0.48^*^	SD < C, L, R
ACE-R Memory (26)	25.0 (1.1)	16.6 (5.8)	19.9 (4.4)	10.6 (5.7)	**H(3) = 30.3, P < 0.0001**	η^2^ = 0.63^*^	L, SD < C SD < L, R
ACE-R Fluency (14)	13.1 (1.2)	8.9 (1.9)	10.9 (2.0)	5.5 (2.9)	**H(3) = 34.6, P < 0.0001**	η^2^ = 0.73^*^	L, SD < C SD < R
ACE-R Language (26)	25.7 (0.5)	21.9 (4.0)	23.4 (1.7)	10.8 (4.2)	**H(3) = 38.2, P < 0.0001**	η^2^ = 0.82^*^	L, SD < C SD < L, R
ACE-R Visuospatial (16)	15.8 (0.6)	15.4 (0.8)	15.6 (0.5)	13.5 (2.8)	**H(3) = 14.9, *P* < 0.01**	η^2^ = 0.28^*^	SD < C
Cambridge Naming (32)	31.9 (0.3)	31.1 (1.2)	31.9 (0.4)	15.7 (7.7)	**H(3) = 37.5, *P* < 0.0001**	η^2^ = 0.80^*^	SD < C, L, R
Boston Naming (30)	29.8 (0.4)	26.0 (2.5)	27.9 (2.3)	8.2 (4.6)	**H(3) = 40.2, P < 0.0001**	η^2^ = 0.87^*^	L, SD < C SD < L, R
Camel and Cactus (32)	30.5 (1.2)	28.6 (1.7)	29.0 (1.6)	16.3 (4.6)	**F(3,18.9) = 44.5, P < 0.0001**	η^2^ = 0.83^*^	SD < C, L, R
Synonym Judgment (48)	47.9 (0.4)	42.8 (1.9)	44.9 (2.9)	36.6 (7.1)	**H(3) = 34.7, P < 0.0001**	η^2^ = 0.74^*^	L, SD < C SD < R
Word-picture matching (36)	35.9 (0.3)	35.8 (0.4)	36.0 (0.0)	32.0 (3.4)	**H(3) = 27.5, P < 0.0001**	η^2^ = 0.57^*^	SD < C, R
Raven’s (12)	10.4 (1.6)	10.5 (1.0)	10.3 (1.9)	8.8 (3.2)	H(3) = 2.0, *P* = 0.58	η^2^ = 0.02^*^	-
Brixton (10)	6.3 (2.2)	7.1 (1.6)	5.7 (2.0)	5.0 (2.9)	H(3) = 4.4, *P* = 0.23	η^2^ = 0.03^*^	-

ACE-R = Addenbrookes Cognitive Examination-Revised, C = control, L = left temporal lobe epilepsy, MMSE = Mini Mental State Examination, R = right temporal lobe epilepsy, SD = semantic dementia, TLE = temporal lobe epilepsy.

Groups compared using Welch ANOVA tests or Kruskal–Wallis tests.

^*^partial η^2^: small = 0.01, medium = 0.06, large = 0.14.

### Person knowledge

In addition to impaired general semantic processing, the SD group displayed a simultaneous degradation of person knowledge. The same pattern was found after unilateral damage, where both TLE groups were impaired on person knowledge, although this was far milder than in SD. There was a significant main effect of group in both the face-name matching (*F*(3,43) = 14.7, *P* < 0.0001, η^2^ = 0.51) and face-profession matching tasks (F(3,43) = 16.0, *P* < 0.001, η^2^ = 0.53). Controls performed better than left TLE (*P* < 0.01), right TLE (*P* = 0.07) and SD (*P* < 0.0001) on the face-name matching task. A similar pattern was found in face-profession matching; SD (*P* < 0.0001), left TLE (*P* < 0.01) and right TLE (*P* < 0.05) groups had poorer scores than controls, and SD were also worse than left TLE (*P* < 0.05).

**Fig. 2:**
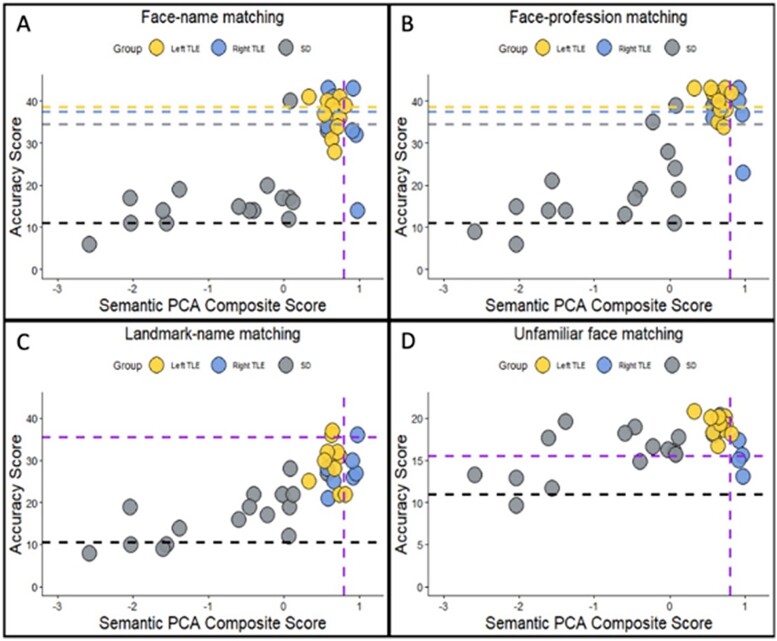
**Neuropsychological performance plotted against semantic PCA composite scores.** (A) Face-name matching. (B) Face-profession matching. (C) Landmark-name matching. (D) Unfamiliar perceptual face matching. The black horizontal line displays chance-level performance. The purple vertical line displays the control lower bound of normality. For (A) and (B) the yellow, blue and gray horizontal lines display the minimum score required in each group to not be impaired if they were the average age of their group (the line color corresponds to the group membership). For (C) and (D) the purple horizontal line displays the score required to not be impaired.

All three patient groups were impaired on the landmark-to-name matching task, demonstrating that the person knowledge deficits found were not selective but generalized to another type of specific-level concept. There was a significant group effect on landmark-name matching (*F*(3,16.7) = 63.7, *P* < 0.0001, η^2^ = 0.77). Post-hoc Games-Howell tests revealed that each patient group performed worse than controls (all *P* < 0.01), and SD performed worse than left and right TLE (both *P* < 0.001). Figure 2 shows performance on each task plotted against semantic composite score for each individual patient. Across each group, most patients were impaired on the face-name matching (percentage impaired; left TLE = 70%, right TLE = 57.1%, SD = 93,8%) and on the difficulty-matched landmark-name matching task (percentage impaired; left TLE = 80%, right TLE = 85.7%, SD = 100%). Fewer patients were impaired on the face-profession matching task (percentage impaired; left TLE = 30%, right TLE = 42.9%, SD = 87.5%). As with general semantic memory, there were no left vs. right differences. No significant differences between left TLE and right TLE were found for face-name matching (*P* = 0.95), face-profession matching (*P* = 0.99), or landmark-name matching (*P* = 0.85).

### Perceptual face matching

#### Accuracy

To explore the impact of bilateral vs. unilateral (left vs. right) ATL damage on perceptual processes, we first examined face matching performance accuracy in the unfamiliar condition only. There was a significant main effect of group on unfamiliar face matching accuracy (*F*(3,43) = 6.8, *P* < 0.001, η^2^ = 0.32), due to poorer performance by the SD group than controls and left TLE (both *P* < 0.01). This effect, however, was driven by a minority of severely impaired SD patients who had the greatest degree of overall semantic impairment (Fig. 2D). Surprisingly, a few of the right TLE patients were also impaired on this task.

**Fig. 3:**
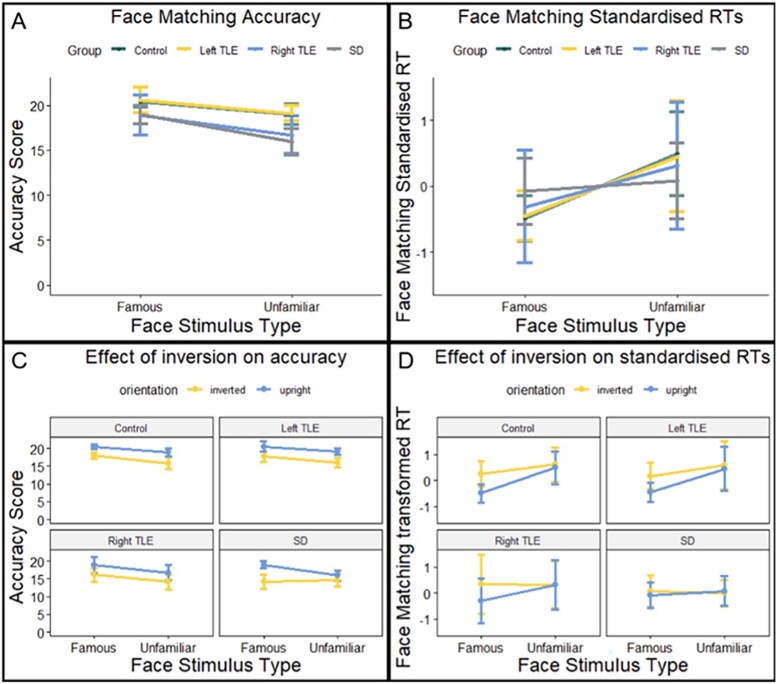
**Mean accuracy and standardized RTs on the perceptual face matching task.** (**A**) Face matching accuracy scores for famous vs. unfamiliar faces. (**B**) Face matching standardized RTs for famous vs. unfamiliar faces. (**C**) Face matching accuracy scores for famous vs. unfamiliar faces in both upright and inverted conditions. (**D**) Face matching standardized RTs for famous vs. unfamiliar faces in both upright and inverted conditions. Bars display 95% confidence intervals.

Next, we further explored the contribution of the ATLs to perception by comparing perceptual face matching performance for famous vs. unfamiliar faces. A familiarity effect was found for all groups—a mixed ANOVA yielded significant main effects of group (*F*(3,43) = 6.8, *P* < 0.001, η^2^ = 0.32) and face stimulus type (*F*(3,43) = 37.8, *P* < 0.0001, η^2^ = 0.47) (Fig. 3A). Each group was more accurate at matching famous faces compared to unfamiliar faces (controls, *P* < 0.001; left TLE, *P* = 0.06; right TLE, *P* < 0.05; SD, *P* < 0.001).

### Response times

As a further assessment of a potential contribution of the ATL to perception, we compared RTs of correct responses to famous vs. unfamiliar faces. To account for differences in baseline RTs between groups, a z-score transformation was applied to the raw RTs (Faust et al. 1999). Raw RTs (Supplementary Fig. 1) were standardized for each participant by taking the RT for each familiarity condition, subtracting the group mean RT (across both conditions) and dividing by the standard deviation of the group RT. This method has been used previously to account for slower baseline responding in SD (Cumming et al. 2006). All groups produced faster responses to famous than to unfamiliar faces, although the effect was reduced in SD and right TLE. A mixed ANOVA revealed an interaction between group and familiarity (*F*(3,43) = 4.0, *P* < 0.05, η^2^ = 0.22) (Fig. 3B). Post-hoc tests revealed that although RTs for famous faces were faster across each group, this difference only reached significance in controls and left TLE (controls, *t* = 4.8, *P* < 0.001; left TLE, *t* = 3.6, *P* < 0.01, right TLE, *t* = 1.5, *P* = 0.18; SD, *t* = 2.0, *P* = 0.07).

### The effect of inversion on perceptual face matching

To explore whether the familiarity effect remained when faces were inverted, three-way mixed ANOVAs were conducted on face matching accuracy and standardized RTs separately, with group, face stimulus type and face orientation as factors. There was a significant three-way interaction between these factors on face matching accuracy (*F*(3,43) = 4.1, *P* < 0.05, η^2^ = 0.22) (Fig. 3C). Inversion abolished the familiarity effect on accuracy in SD but not in patients with unilateral damage or in healthy controls. Separate ANOVAs on each group revealed a two-way interaction between face stimulus type and orientation in SD (*F*(1,15) = 9.5, *P* < 0.01, η^2^ = 0.39), but not in controls (*F*(1,13) = 0.6, *P* = 0.47, η^2^ = 0.04), left TLE (*F*(1,9) = 0.04, *P* = 0.84, η^2^ = 0.005) or right TLE (*F*(1,6) = 0.03, *P* = 0.87, η^2^ = 0.005). There was no three-way interaction between group, face stimulus type and face orientation on standardized face matching RTs (*F*(3,43) = 1.0, *P* = 0.39, η2 = 0.07). There were significant two-way interactions between group and face stimulus type (*F*(3,43) = 5.9, *P* < 0.01, η^2^ = 0.29), group and face orientation (*F*(3,43) = 3.4, *P* < 0.05, η^2^ = 0.19), and face stimulus type and face orientation (*F*(3,43) = 22.8, *P* < 0.0001, η^2^ = 0.35) (Fig. 3D).

### RTs across different levels of person knowledge

**Fig. 4:**
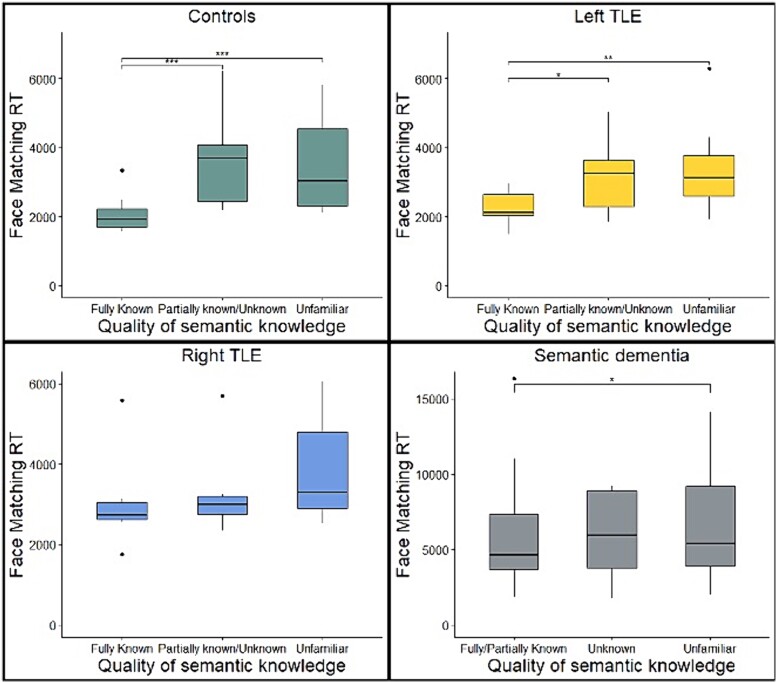
**Perceptual face matching RTs for different levels of semantic knowledge.** (**A**) Controls, (**B**) left TLE, (**C**) right TLE, and (**D**) semantic dementia. ^*^P < 0.05, ^*^^*^P < 0.01, ^*^^*^^*^P < 0.001.

The correspondence between item-specific semantic status and perceptual performance was assessed by categorizing face matching trials into “fully known,” “partially known” or “unknown,” based on semantic performance in the person knowledge tasks. This method allows perceptual performance to be compared across items and has been used in previous studies of visual recognition in SD (Graham et al. 2000; Simons et al. 2001). As there were very few “unknown” trials in the controls and TLE groups, the “unknown” and “partially known” trials were combined into a single category. Similarly, “fully known” and “partially known” were combined in the SD group due to a lack of “fully known” trials. In all groups, face matching RTs were quicker for “fully known” trials (i.e. with the most semantic knowledge), further highlighting the association between semantic knowledge and perception (Fig. 4). Friedman tests revealed a main effect of semantic knowledge in controls (χ^2^(2) = 19.8, *P* < 0.0001, W = 0.76), with faster RTs for “fully known” items compared to “partially known”/“unknown” (*P* < 0.001) and unfamiliar items (*P* < 0.001). There was also a main effect of semantic knowledge in left TLE (χ^2^(2) = 12.6, *P* < 0.01, W = 0.63) with faster RTs for “fully known” than “partially known”/“unknown” (*P* < 0.05) and unfamiliar items (*P* < 0.01). There was a significant main effect in the SD group also (χ^2^(2) = 6.9, *P* < 0.05, W = 0.23), with faster RTs for “fully/partially known” compared to unfamiliar items (*P* < 0.05). Surprisingly, there was no effect of semantic knowledge on RTs in the right TLE group (χ^2^(2) = 2.6, *P* = 0.28, W = 0.18).

## Discussion

Parallel theories and literatures have been developed linking the ATL with various cognitive functions including face recognition, semantic knowledge and social cognition, each supported by evidence from neuropsychology, functional neuroimaging and other techniques (Olson et al. 2013; Collins and Olson 2014a; Lambon Ralph et al. 2017). This study focused on two prominent branches of the literature—(i) face recognition and (ii) semantic memory. The discovery that the ATLs respond to familiar faces and that ATL damage causes face recognition deficits has led to proposals that the ATLs should be considered part of an extended face processing network (Haxby et al. 2000; Gobbini and Haxby 2007; Collins and Olson 2014a; Duchaine and Yovel 2015). A separate substantial literature proposes that the ATLs are crucial to the formation and activation of all concepts and at all “levels” from superordinate (e.g. animals or objects), to intermediate (e.g. humans or buildings) to specific (e.g. Marilyn Monroe, the Eiffel Tower), with increasing demands on ATL processing along this succession of levels (Rogers et al. 2015). The global aim of this investigation was to bring these two parallel literatures together, via both general semantic and face processing tasks applied to patients with two different types of ATL damage: bilateral atrophy in SD vs. unilateral resection in treatment for epilepsy.

Although most of the SD cases had, as is typical, a degree of ATL asymmetry, the distribution of volume loss was clearly bilateral in contrast to the unilateral loss following resection. Consequently, we were able to explore the impact of bilateral vs. unilateral (left or right) ATL damage on (i) general semantic memory, (ii) person knowledge, and (iii) the perceptual processes that are primarily considered to be functions of ventral occipitotemporal areas. In the following sections we integrate the key findings of the study within a unified neurocognitive framework for the ATLs in face recognition, person knowledge and semantic memory, and discuss the implications for the extended face network (Haxby et al. 2000; Gobbini and Haxby 2007; Duchaine and Yovel 2015).

*1: The ATLs support a singular, common semantic system*

Bilateral ATL damage generated substantial impairments in both general semantic processing and person knowledge. The semantic impairment occurred for all types of stimuli and across expressive and receptive tasks, in line with the global degradation of conceptual knowledge characteristic of SD (Bozeat et al. 2000; Mummery et al. 2000). This study highlighted the consequences of bilateral ATL damage on person knowledge, supporting previous neuropsychological investigations in SD (Snowden et al. 2004, 2012). Unilateral ATL damage also caused dual impairments in general semantic memory and person knowledge, although to a much milder degree than the bilateral damage in SD. This finding mirrors previous studies of ATL-resected patients, which have reported a subtle generalized semantic impairment following resection of either the left or right ATL (Wilkins and Moscovitch 1978; Lambon Ralph et al. 2012; Rice et al. 2018a). Taken together, the results from the two patient groups are consistent with a semantic system underpinned by the bilateral ATLs that represents all types of conceptual knowledge, including person knowledge (Patterson et al. 2007; Lambon Ralph et al. 2017).

Knowledge of famous people was severely impaired by bilateral ATL damage, and many SD patients performed around chance-level on the tasks assessing this cognitive sphere. Bilateral damage caused a similarly severe deficit in a landmark knowledge task, which was included as it taps into another type of specific-level concept/“unique entity” exemplar (Grabowski et al. 2001; Ross and Olson 2012). Neuropsychological studies have demonstrated that the semantic decline in SD is graded, such that specific-level individuations (e.g. differentiating between a dalmatian and other breeds of dog) are more vulnerable than more basic semantic distinctions (e.g. differentiating between a dalmatian and other types of mammal) (Warrington 1975; Hodges et al. 1995; Rogers et al. 2006; Rogers et al. 2015). Consequently, tasks requiring specific-level distinctions are the most sensitive assessments of semantic integrity (Rogers et al. 2006). Clear impairments for the specific-level concepts were also found after unilateral damage (although much milder than in SD), in line with previous findings that the semantic deficits from unilateral ATL damage are amplified when more challenging tasks or concepts are used (Lambon Ralph et al. 2010; Lambon Ralph et al. 2012; Rice et al. 2018a). The results here indicate therefore that, although all concepts are supported by the ATLs, the representations in the semantic system are such that specific-level concepts (of which individual people or landmarks are examples) are inherently more vulnerable to mild damage (as simulated in multiple implemented computational instantiations of the hub-and-spoke model) (Rogers et al. 2004a; Chen et al. 2017).

*2: The functionally-unitary semantic system is supported bilaterally*

There were no selective semantic deficits after either left or right unilateral ATL damage: both were characterized by a mild generalized semantic impairment. This finding implicates the bilateral ATLs as important for conceptual knowledge, a proposal which is supported by convergent evidence from studies in patients and in healthy participants. FMRI studies consistently detect bilateral ventrolateral ATL activation when healthy participants engage in semantic processing (provided appropriate techniques are used to maximize the otherwise “shy” ventral ATL signal) (Binney et al. 2010; Humphreys et al. 2015). Furthermore, local field potentials in overlapping bilateral ventrolateral ATL regions have been detected from grid electrode recordings during semantic tasks in preresected patients (Shimotake et al. 2015). Causal evidence for the ATLs in semantic memory has also been derived from neurostimulation studies: both TMS to either the left or right ATL in healthy participants (Lambon Ralph et al. 2009) and direct cortical stimulation of the left or right ventrolateral ATL (Shimotake et al. 2015) produce a transient slowing of semantic processing but not nonsemantic processing. There is evidence that functional connectivity between the ATLs increases during challenging semantic tasks in healthy participants with the degree of functional connectivity predicting semantic performance (Jung et al. 2021) as well as behavioral outcome after stroke (Warren et al. 2009). Consequently, it appears that the ATLs work together as a single semantic system, where both the (i) integrity of the left and right ATL and the (ii) functional connectivity between the ATLs are crucial.

Despite similarities in the *quality* of the semantic impairment, there were differences in the *magnitude* of the impairment from bilateral vs. unilateral ATL damage. The finding of mild impairment after unilateral damage vs. severe deficits after bilateral damage mirrors previous neuropsychological investigations (Bozeat et al. 2000; Lambon Ralph et al. 2012) and also fits with the classical comparative neurology literature, where bilateral ATL ablation in macaques (and in one human case) generates a severe multimodal associative agnosia, yet unilateral resection yields only a mild and transient effect (Klüver and Bucy 1939; Terzian and Ore 1955). Strikingly, there was considerable overlap in total ATL damage across the two groups, meaning that, whilst the level of semantic impairment is governed by the overall level of ATL damage (Ding et al. 2020; Ramanan et al. 2023), the uni−/bi-lateral distribution of damage is also crucial.

The findings described above are in line with the bilateral distribution of semantic representations across the left and right ATL. One advantage of a bilateral-implementation is that it makes the semantic system more robust to unilateral damage. Building on early work by Lambon Ralph et al (Lambon Ralph et al. 2001), this hypothesis was captured and formally explored in formal computational models (Schapiro et al. 2013) in which the single hub-and-spoke semantic model (Rogers et al. 2004a) was split into two partially interconnected “demi-hubs” (mimicking the left and right ATLs). Simulated unilateral damage generated a much milder impairment than bilateral damage, even when total damage was kept constant. After unilateral damage, the undamaged contralateral demi-hub was able to function with higher accuracy albeit more slowly than before (Schapiro et al. 2013). Formal analyses of this model showed that there were two causes of this difference. One factor is some redundancy of representation across the two ATL “demi-hubs.” The other is that, when a system is damaged, not only is the representation weakened but it also becomes noisier, which can be propagated to connected units. Thus, after bilateral damage this noise percolates throughout, whereas unilateral damage tends to restrict the resultant noise just to the damaged side. In addition (following Lambon Ralph et al. 2001), differential connectivity of the left and right ATL hubs to other systems (e.g. speech output) gives rise to the known differences (e.g. left ATL damage produces greater levels of anomia). Additional insights into the compensatory neural mechanisms underlying the ATL’s resilience to unilateral damage/perturbation have been derived from fMRI studies, where, after unilateral damage/perturbation (either from resection or rTMS), the unaffected contralateral ATL not only upregulates its activity but increases its effective connectivity with the affected ATL (Binney and Lambon Ralph 2015; Jung and Lambon Ralph 2016; Rice et al. 2018b).

Bilateral implementation may be a property of other brain regions beyond the ATL. For example, bilateral hippocampal removal generates a catastrophic dense amnesia (Scoville and Milner 1957; Penfield and Milner 1958), whereas unilateral resection yields mild episodic memory deficits (Bell and Davies 1998). This neuropsychological pattern is redolent of the semantic memory literature, and implies that bilateral organization may be a more general neurocomputational principle (Schapiro et al. 2013).

*3: The ATL-semantic system interacts with posterior temporal regions to support face perception*

Although the ATL is critical for semantic memory, there was no evidence that this region is similarly critical for the ability to discriminate between faces based on visual properties. Face perception abilities were preserved after either bilateral or unilateral ATL damage (except for a minority of severe SD patients). This result aligns with previous findings of intact face perception abilities alongside a preservation of perceptual skills broadly in SD (Neary et al. 1998; Gorno-Tempini et al. 2011; Hutchings et al. 2017; Borghesani et al. 2019). The few SD patients who were impaired at unfamiliar face matching had the lowest composite semantic scores, implying increased levels of disease severity. Perceptual deficits in these patients may therefore be explained by the spread of atrophy into posterior temporal regions critical for face perception (e.g. fusiform face area) rather than from ATL damage per se.

The contribution of ATL-based semantics to face perception was explored by assessing the classic face familiarity effect (Bruce 1986; Young et al. 1986). The familiarity effect was robustly replicated in healthy participants, even when faces were inverted (although, as expected in this classic paradigm, to a lesser degree than upright). In contrast to the patients’ preserved face-matching abilities, bilateral ATL damage and associated semantic degradation diminished the familiarity effect. The relationship between semantic knowledge and face perception was further highlighted by the finding of item-specific correspondence between the quality of semantic knowledge and the strength of the familiarity effect, across all participant groups. Inversion completely obliterated the familiarity effect in bilateral ATL cases, which implies that the semantic contribution to face perception is maintained when faces are inverted but is more subtle and thus more sensitive to semantic degradation.

Although it was not possible from our data to provide direct evidence, we speculate that the familiarity effect reflects interactivity between the ATL and ventral occipitotemporal cortex such that during perception of famous faces, the activated semantic system feeds back expectations/predictions about the input to support the early stages of visual processing. Bilateral ATL damage would result in degraded and diminished semantic representations being projected back to posterior perceptual areas, thus disrupting any facilitation or acceleration that is provided by a healthy semantic system. This proposal can be accommodated within the hub-and-spoke model of semantic memory where, through its interactivity and connectivity, the ATL-semantic hub not only receives inputs from modality-specific posterior areas but also projects back to them (Rogers et al. 2004a).

In line with this proposal, depth electrode recordings have detected initial “first-pass” activation in the ATLs during visual recognition, which then may feedback activated semantics to posterior temporal cortex (Chan et al. 2011). In addition, there is electrophysiological evidence that semantic information modulates ERPs associated with early visual processing (Abdel Rahman and Sommer 2008; Heisz and Shedden 2009; Herzmann and Sommer 2010; Abdel Rahman and Sommer 2012). Further evidence for an interaction between conceptual and perceptual systems derives from people with SD who are impaired on perceptual tasks such as object recognition (Ikeda et al. 2006), word recognition (Cumming et al. 2006) and object/lexical decision (Rogers et al. 2004b) with the perceptual impairment aligning with the level of semantic degradation. Most strikingly, when SD patients are asked to copy line drawings of real objects/animals a mere 10 s after the stimulus pictures have been withdrawn, their degraded semantic systems delete item-specific features (e.g. a camel’s hump) and include properties that are true more generally of that class but not of the specific concept just presented (e.g. drawing a duck with four legs) (Bozeat et al. 2003).

*4. Graded functional differences between the ATLs emerge through different connectivity strengths with modality-specific regions*

Although there were no significant differences in semantic performance after left vs. right ATL resection, people with unilateral right ATL damage performed more poorly at perceptual face matching than their left-sided counterparts, in terms of reduced accuracy and a diminished familiarity effect. Face recognition problems have previously been reported after right ATL damage from unilateral resection for TLE (Seidenberg et al. 2002; Glosser et al. 2003;Drane et al. 2013 ; Rice et al. 2018a) and also right > left ATL atrophy in SD (Evans et al. 1995; Joubert et al. 2006). Previous research has found left ATL damage to be associated with deficits in naming famous people (Glosser et al. 2003; Drane et al. 2013; Borghesani et al. 2019) whereas right ATL damage has been linked to deficits in familiarity judgments (Borghesani et al. 2019) and retrieval of nonverbal semantic information about people (Snowden et al. 2004, 2012).

According to the hub-and-spoke model, although conceptual knowledge is represented bilaterally, graded asymmetries may emerge from different connectivity strengths of the left and right ATL with modality-specific regions. As a result, although all aspects of semantic memory would be impaired by ATL damage, some types of semantic task may be disproportionately affected if the damage is asymmetric (Rice et al. 2015a; Lambon Ralph et al. 2017; Woollams and Patterson 2018). The most reliable example is anomia, which is more severe after left ATL damage in both SD (Lambon Ralph et al. 2001; Woollams and Patterson 2018) and unilateral resection for TLE (Drane et al. 2013; Rice et al. 2018a). The increased anomia from left ATL damage has been attributed to the region having stronger connections with left-lateralized speech production areas, a proposal which has been captured computationally (Lambon Ralph et al. 2001).

There is a right-sided dominance for face processing in the posterior ventral temporal cortex (Kanwisher et al. 1997; Behrmann and Plaut 2015; Hoffman and Lambon Ralph 2018). In the posterior ventral temporal cortex, there are graded asymmetries in functional organization rather than absolute differences between the hemispheres, such that face processing is supported bilaterally but more strongly in the right hemisphere (Plaut and Behrmann 2011; Behrmann and Plaut 2015). The increased face recognition problems after right sided ATL damage might reflect downstream effects of this functional asymmetry, i.e. the stronger visual input from the right posterior temporal cortex is projected to the right ATL (Hoffman and Lambon Ralph 2018).

Relative specializations *within* the ATLs may also emerge via the same principle of graded connectivity (Visser and Lambon Ralph 2011; Binney et al. 2012; Rice et al. 2015a; Lambon Ralph et al. 2017). For example, there is fMRI evidence that, in addition to activation in a core ventrolateral ATL “hotspot,” person knowledge (faces and written names of famous people) elicits weaker yet selective activation in a slightly anterior ATL subregion (Rice et al. 2018c). The temporal poles are most strongly connected to the orbitofrontal cortex via the uncinate fasciculus (Papinutto et al. 2016) leading to speculation that the relative preference of this ATL subregion for person knowledge reflects its proximity to paralimbic regions, which may represent “spokes” particularly important for the formation of person knowledge (Rice et al. 2018c; Riberto et al. 2019).

### Implications for the extended face network

Our findings have three key implications for the extended face network. First, the core function of the ATL in face recognition is the representation of semantic memory. Damage to the ATL does not impair the perceptual processes necessary for face perception, which instead depend on “core” face recognition areas in more posterior temporal regions (Barton et al. 2002; Barton 2008). Rather, ATL damage degrades the semantic representations which are needed to support familiar face recognition through the provision of activated semantics. Critically, the ATLs are not face-selective, but support person knowledge as part of a transmodal semantic representational system.

Second, the extended network is interactive in nature. Rather than a purely feedforward hierarchical ventral pathway, the core posterior temporal face perception areas interact bidirectionally with ATL-semantic regions (that code information about people—not just faces, alongside all other concepts). Accordingly, activated semantics project back expectations/predictions about the input to support the early stages of visual processing via rapid feedback along the inferior longitudinal fasciculus. Following this semantic feedback from the ATL, perceptual demands are reduced when faces are familiar, which leads to a boost or acceleration of recognition.

Third, the extended face network recruits the ATLs bilaterally. Existing models of face recognition have not made strong claims on the differential roles of the left/right ATL in the extended face recognition network (Haxby et al. 2000; Gobbini and Haxby 2007; Duchaine and Yovel 2015), although the nature of the discussion about core areas of the face recognition network is itself predominantly “right-lateralized” (Kanwisher et al. 1997; Ishai et al. 2005; Rossion et al. 2012; Behrmann and Plaut 2015). In this study we demonstrated that person knowledge is supported by the left and right ATL, as part of a broader conceptual representational system. However, the right ATL may be relatively more important for face recognition because it receives increased visual input from right posterior temporal ventral cortex.

### Limitations

SD and ATL-resected TLE are clinical entities associated with distinct aetiologies and neuropathologies (progressive neurodegeneration versus resection to treat drug-resistant epilepsy) meaning there are important factors to consider when making direct comparisons. In contrast to SD, where ATL damage occurs in the context of a previously intact and typically organized semantic system, chronic TLE raises the possibility of presurgical changes in functional organization (Goldmann and Golby 2005). Indeed, there is evidence of functional and structural connectivity alterations to language networks in TLE (Powell et al. 2007; Trimmel et al. 2018; Trimmel et al. 2021). According to this “functional re-organization” hypothesis, the minimal impact of resection on semantic memory in TLE might occur because the ATL is no longer supporting this function in these participants. Studies have found that functional re-organization in TLE is associated with a young-onset of epilepsy, presumably due to the greater capacity for plasticity in maturing brains (Duchowny et al. 1996; Goldmann and Golby 2005; Trimmel et al. 2018). As the TLE participants in the current study were all adult-onset, this implies an increased likelihood of typical presurgical brain organization in our cohort. Furthermore, there is evidence to suggest that the bilateral ATLs remain as core semantic regions in presurgical TLE. Grid electrode studies in presurgical TLE have found neural activity in the left and right ATLs during semantic processing, in exactly same region that shows fMRI activation for semantic tasks in healthy participants (Binney et al. 2010), while direct stimulation of these regions generates a transient semantic impairment in TLE patients (Shimotake et al. 2015; Rogers et al. 2021).

## Conclusion

Taken together, our results demonstrate that the bilateral ATL is critical for general semantic memory and person knowledge. Perceptual face matching performance is preserved following ATL damage, however bilateral ATL damage diminishes the familiarity effect, which suggests that conceptual knowledge interacts with perceptual processes to support face recognition. Our findings converge on a model of the bilateral ATLs as a functionally-unitary transmodal semantic hub, which supports face perception through the provision of activated semantics.

## Supplementary Material

Supplementary_data_bhae336
