# Supplementary material for: The impact of bilateral versus unilateral anterior temporal lobe damage on face recognition, person knowledge and semantic memory
Source: Cereb Cortex. 2024 Aug 10;34(8):bhae336. doi: 10.1093/cercor/bhae336 (PMC11315654; doi:10.1093/cercor/bhae336)
Supplement: Supplementary_data_bhae336 [file supplementary_data_bhae336.docx]

Supplementary Table 1. Voxel based morphometry results.

| Regions | Hemisphere | Number of voxels | Peak MNI co-ordinate | | | Peak MNI co-ordinate region | *t*-value |
| --- | --- | --- | --- | --- | --- | --- | --- |
|  |  |  | *x* | *y* | *z* |  |  |
| *Left TLE < Controls* | | | | | | | |
|  | Left | 12,093 | -44 | 9 | -42 | Temporal pole | 18.5 |
| *Right TLE < Controls* | | | | | | | |
|  | Right | 16,772 | 35 | -2 | -48 | Inferior temporal gyrus | 29.8 |
| *SD < Controls* |  |  |  |  |  |  |  |
|  | Left | 21,630 | -33 | 8 | -29 | Temporal pole | 17.0 |
|  | Right | 6,810 | 33 | 6 | -27 | Temporal pole | 11.8 |
|  | Left | 685 | -53 | -53 | 3 | Middle temporal gyrus | 8.9 |

MNI = Montreal Neurological Institute, SD = semantic dementia, TLE = temporal lobe epilepsy. Clusters reported if significant at *P*(FWE) < 0.05 with a cluster threshold of 100 voxels

Supplementary Table 2. P-values for post-hoc tests

|  | C v L | C v R | C v SD | L v R | L v SD | R v SD |
| --- | --- | --- | --- | --- | --- | --- |
| ACE-R total* | < 0.01 | < 0.05 | < 0.0001 | ns | < 0.001 | < 0.0001 |
| MMSE† | < 0.01 | ns | < 0.0001 | ns | Ns | < 0.05 |
| ACE-R Attention† | ns | ns | < 0.0001 | ns | < 0.05 | < 0.01 |
| ACE-R Memory† | < 0.01 | ns | < 0.0001 | ns | ns | < 0.05 |
| ACE-R Fluency† | < 0.01 | ns | < 0.0001 | ns | ns | < 0.01 |
| ACE-R Language† | < 0.05 | ns | < 0.0001 | ns | < 0.05 | < 0.05 |
| ACE-R Visuospatial† | ns | ns | < 0.01 | ns | ns | ns |
| Cambridge Naming† | ns | ns | < 0.0001 | ns | < 0.001 | < 0.001 |
| Boston Naming† | < 0.05 | ns | < 0.0001 | ns | < 0.05 | < 0.01 |
| Camel and Cactus* | < 0.05 | ns | < 0.0001 | ns | < 0.0001 | < 0.0001 |
| Synonym Judgement† | < 0.01 | ns | < 0.0001 | ns | ns | < 0.05 |
| Word to Picture Matching† | ns | < 0.01 | < 0.0001 | ns | < 0.001 | ns |

ACE-R = Addenbrooke’s Cognitive Examination-Revised, C = control, L = left TLE, MMSE = Mini Mental State Examination, ns = not significant, R = right TLE, SD = semantic dementia

*Games-Howell test

†Dunn test (Holm-corrections)


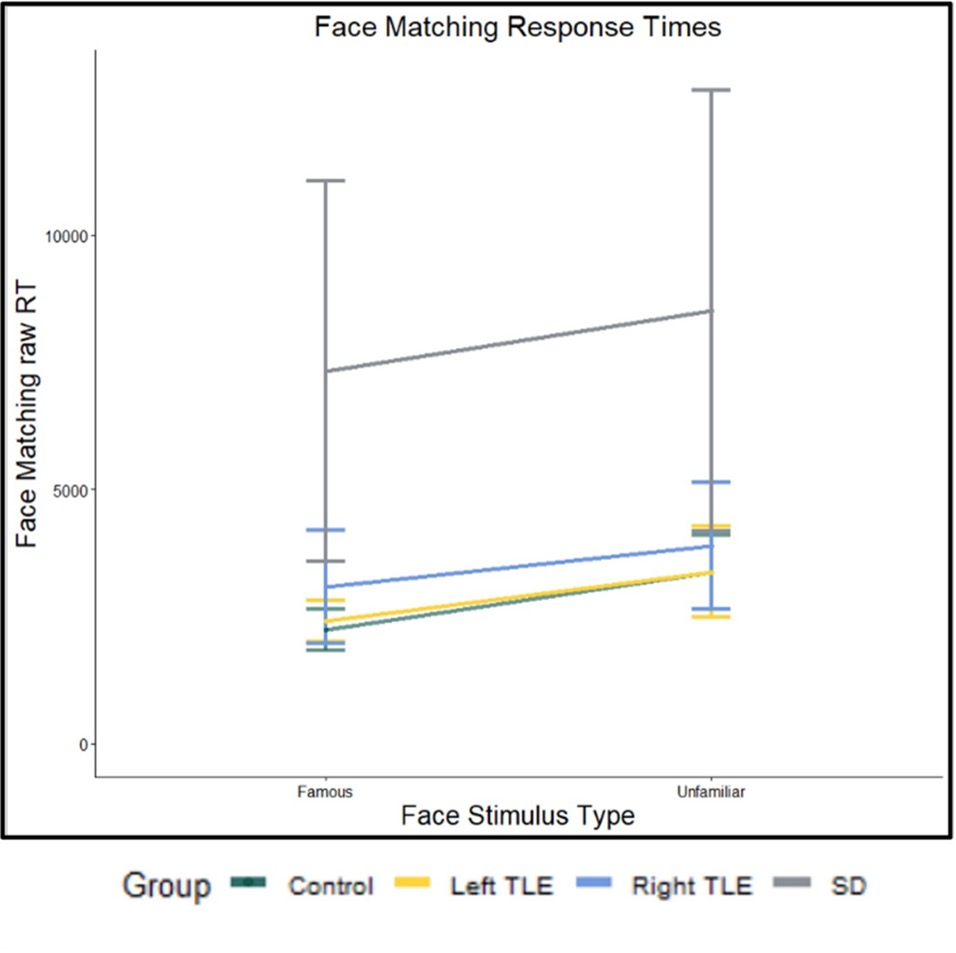


Supplementary Figure 1. Raw RTs on the perceptual face matching task. Bars display 95% confidence intervals.
